# Ownership Change and Care Quality: Lessons From Minnesota’s Experience With Value-Based Purchasing

**DOI:** 10.1093/geroni/igac022

**Published:** 2022-04-09

**Authors:** Zachary Hass, Kathleen Abrahamson, Greg Arling

**Affiliations:** School of Nursing, Purdue University, West Lafayette, Indiana, USA; School of Industrial Engineering, Purdue University, West Lafayette, Indiana, USA; Regenstrief Center for Healthcare Engineering, Purdue University, West Lafayette, Indiana, USA; School of Nursing, Purdue University, West Lafayette, Indiana, USA; Center for Aging and the Life Course, Purdue University, West Lafayette, Indiana, USA; School of Nursing, Purdue University, West Lafayette, Indiana, USA; Center for Aging and the Life Course, Purdue University, West Lafayette, Indiana, USA

**Keywords:** Health care policy, Nursing homes, Person-centered care, Quasiexperimental design

## Abstract

**Background and Objectives:**

Minnesota’s implementation of a new nursing home value-based reimbursement (VBR) system in 2016 presented an opportunity to compare the response of nursing homes (NHs) to financial incentives to improve their quality and efficiency. The state substantially increased reimbursement for care-related costs and tied this rate increase to a composite quality score. Coinciding with rate increases of the new VBR system was an increase in ownership changes, with new owners being primarily for-profit entities from outside of Minnesota, including several private equity firms. Our objective was to examine NHs that underwent a change in ownership to determine their cost and quality response to the change.

**Research Design and Methods:**

Our sample consists of 342 Minnesota NHs that submitted Medicaid cost reports each year from 2013 to 2019. A time differential two-way fixed-effects difference-in-difference model is used to assess changes in quality metrics by comparing measures in years prior to and years following the sale for NHs that changed ownership versus NHs with consistent ownership. Nursing home characteristics, revenue, and spending patterns are examined to understand differences in performance.

**Results:**

Those NHs with ownership change experienced a decline in quality scores with notable changes to expenditure patterns. They performed worse on Minnesota Department of Health inspection scores and had nonsignificant declines in measures of quality of life and clinical care. They had declining staff dental and medical benefits and occupancy rates, greater revenue growth from Medicare Part B, and larger increases in administrative management fees.

**Discussion and Implications:**

Minnesota like many other states has given wide latitude for nursing home ownership changes, without specific oversight for the quality of care and expenditure patterns of new owners. Recommendations include strict guidelines for the transparency of ownership structures, quality performance targets, rigorous financial auditing, and enhanced regulatory oversight.

**Translational Significance:** With the increasing interest of profit-seeking investors in NHs, it is important that regulators establish sufficiently strong mechanisms of accountability that properly incentivize care quality. Potential mechanisms are setting a minimum quality threshold for new owners, strengthening the link between quality measures and reimbursement rates, and establishing a policy to deny a change in ownership based on a poor performance record of the acquiring owner.

Nursing homes (NHs) in the United States have a variety of ownership structures. Existing research demonstrates that ownership type influences NH quality. For-profit ownership has been correlated with lower quality post-acute care (Grabowski et al., 2013), lower nurse staffing levels, and higher regulatory deficiencies than nonprofit ownership (Harrington et al., 2012). Two systematic reviews (Comondore et al., 2009; Hillmer et al., 2005) concluded that quality of care delivered within for-profit NHs was generally lower than quality of care within nonprofit NHs. However, multiple methodological limitations are present in this body of research, including primarily cross-sectional designs and lack of a standardized definition/measure of care quality.

As of 2020, 70% of U.S. NHs were owned by for-profit entities, 23% by nonprofit entities, and 6% by government agencies (Distribution of Certified Nursing Facilities by Ownership Type, 2021). Over the past decade, ownership type in the United States has remained relatively stable in regards to the for-profit/nonprofit dichotomy: in 2010, 68% of NH were for-profit, 26% nonprofit, and 6% government owned (Harrington et al., 2011). Much of the existing research examines difference in quality between for-profit/ nonprofit ownership types. However, examining the for-profit/nonprofit difference may obfuscate trends that occur within each designation, particularly within the for-profit sector. For-profit owners may take a number of configurations, from an individual to a large corporate chain. Private equity investment firms own about 11% of U.S. NHs (Gupta et al., 2021). Private equity ownership is of particular interest due to its rapid growth in investment in recent years; $5.3 billion dollars in NH investment since 2015 compared with $1 billion between 2010 and 2014 (Harrington et al., 2021), and implications for quality given the often distant and disengaged investors with a motivation toward short-term profits and growth (Gupta et al., 2021).

Recent studies have had mixed findings regarding private equity ownership and quality. Braun et al. (2020) found no significant difference in COVID-19 cases or deaths between private equity owned NHs and other ownership types in the United States. Using data from Ohio, Huang and Bowblis (2019) concluded that private equity ownership does not result in lower care quality for long stay NH residents. Conversely, Gupta et al. (2021) discovered stark differences when comparing private equity outcomes to those of other ownership types among a sample of 18,485 U.S. NHs between 2000 and 2017. Residents within private equity owned NHs were 10% more likely to die during their stay or 90 days after, 50% more likely to take an antipsychotic medication, and have 3% less direct care aides to assist with daily needs. Gupta et al. (2021) also found that the amount billed per 90-day care episode was 11% higher for private equity residents, undermining the supposed cost-efficiency benefit of these NHs.

Because state policy applies a strong influence over the regulation and financing of NH care, ownership type and financial motivation to operate a NH is affected by state context. States vary widely in for-profit NH ownership, as well as private equity ownership. For example, 84% of NHs in California are operated for-profit, in comparison to 4% in North Dakota (Distribution of Certified Nursing Facilities by Ownership Type, 2021). Minnesota, at 31%, is among the states with the lowest percentage of for-profit ownership, with lower rates only in Alaska (15%) and North Dakota (4%). Braun et al. (2020) identified 128 private equity acquisitions in Ohio between 2010 and 2020, while noting only 10 in New York. As we note below, Minnesota had a substantial growth in private equity acquisitions during this period.

In 2016, Minnesota implemented a value-based reimbursement (VBR) policy for Medicaid payments to NHs. One of the goals of VBR was to incentivize improvement of NH quality as measured by a composite quality score that includes measures of quality of life and clinical care quality. By attempting to tie care quality to reimbursement rates, VBR was part of the broader trend in value-based payments in healthcare to incentivize care quality and person-centered care (Briesacher et al., 2009; Burwell, 2015; Conrad, 2015).

A second goal of VBR was to increase NH cost coverage for Medicaid-enrolled NH residents. By virtue of the state’s rate equalization policy, which requires private pay rates to remain equal to Medicaid rates, costs for privately paying residents would also increase. Upon implementation of rate changes Minnesota had a marked rise in the number of annual NH sales or changes of ownership (CHOW). From 2014 to 2019, 80 NHs were sold, representing approximately 23% of Minnesota NHs that were in continuous operation over the period. These rapid changes in ownership were driven, at least in part, by national trends in the NH industry of increased ownership of NHs by private equity firms and the separation of physical plant ownership and operation through the vehicle of real estate investment trusts (Geyman, 2021; Harrington et al., 2017). In addition to being likely to engage in CHOWs, large for-profit NH chains and those owned by private equity firms have had a history of poor care quality and regulatory violations (Braun et al., 2020; Grabowski et al., 2013; Harrington et al., 2012; Hirth et al., 2014).

Our study is motivated by the absence in the research literature of well-established findings in regard to the impact of ownership on NH quality. Little research has been conducted longitudinally to examine care quality of NHs before and after the ownership conversion. Instead, studies have examined point in time associations between ownership and care quality measures. Additionally, we are interested in the impact of ownership changes within the context of a VBR policy implementation. Better understanding of the relationship between CHOW and quality performance can inform reimbursement design, so that policy makers can incentivize improved care quality of new owners that are attracted by VBR.

## Study Objective

The objective of the current study is to determine the impact of NH ownership change on care quality and expenditures within the context of VBR policy implementation in Minnesota.

## Method

### Data

Data were derived from annual cost reports submitted by NHs to the Minnesota Department of Human Services and from the Minnesota Nursing Home Report Card (Nursing Home Report Card, 2021). The cost reports contain NH characteristics, staffing patterns, and expenditures. The quality score data contain the three subcomponent scores used in the original quality score tied to the quality incentive of the VBR policy. The total quality score is scaled to 100 points and is made up of a clinical quality indicator score (scaled to 50 points), a quality of life score (scaled to 40 points), and a Minnesota Department of Health inspection score (scaled to 10 points). The clinical quality indicator score is derived from 19 long stay and 2 additional short stay measures found in the Minnesota Nursing Home Report Card. The measures from the Minimum Data Set cover behavior, depression, restraints, continence, infection, falls, nutrition, skin care, psychotropic drug use, physical functioning, and pain management (Nursing Home Report Card, 2021; Xu, 2021). The quality of life score is derived from resident surveys conducted by an independent research firm and covers the domains of meaningful activities, food enjoyment, environment, dignity, autonomy, relationships, caregiving, and mood (Nursing Home Report Card, 2021; Rurka & Xu, 2021). The Minnesota quality measures overlap to some extent the measures in Medicare’s Nursing Home Compare (Medicare.gov, 2022). However, the Minnesota system predates Nursing Home Compare, has additional indicators of care quality, and employs more extensive risk-adjustment. The state developed these measures with stakeholder input on operational definitions, implementation, and reporting. Data on NH ownership came from state administrative records.

### Analysis

The analysis uses a time differential two-way fixed-effects difference-in-difference model (Cunningham, 2021) examining the overall quality score and the three subcomponent measures for change in quality scores from the years before to after the change of ownership relative to NHs with constant ownership. The model estimates a weighted average of all possible 2 × 2 difference-in-difference estimates between CHOW and non-CHOW NHs from each event year (Goodman-Bacon, 2021).The total sample consisted of 342 Minnesota NHs that submitted cost reports each year from 2013 to 2019. Of these, 80 NHs changed ownership between 2014 and 2019 with the highest number of changes of ownership occurring in 2017 (26 NHs) and lowest in 2014 (5 NHs).

**Figure 1.**
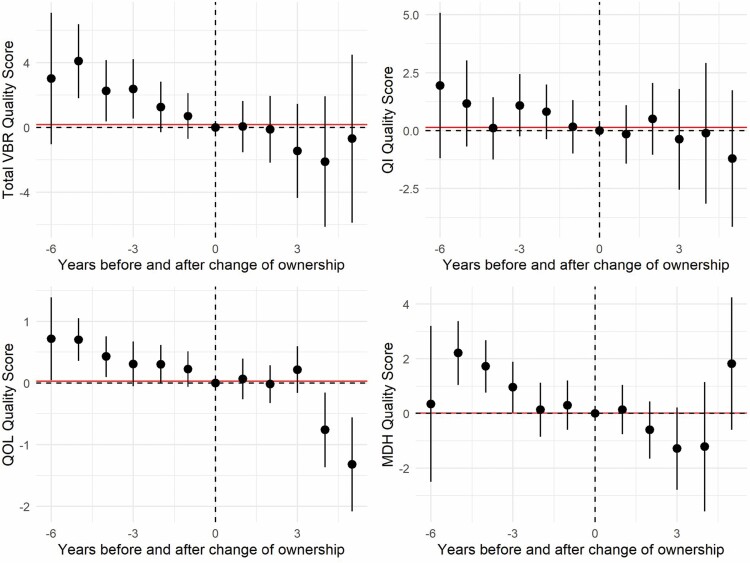
Event study graphs of quality score deviations near year of ownership change. Number of facilities by years prior to changes of ownership (CHOW) (lead) and years after CHOW (lag)—lead 6: 10, lead 5: 29, lead 4: 55, lead 3: 68, lead 2: 75, lead 1: 80, lag 1: 70, lag 2: 51, lag 3: 25, lag 4: 12, lag 5: 5.

Event study graphs were used to visualize trends in quality scores within those facilities that changed ownership relative to the year of sale (Cunningham, 2021). To produce these graphs, regression models were fit with the total quality score or one of the subcomponents as the response, dummy variables for time relative to the change of ownership (baseline), cost year, and facility fixed effects as the independent variables. Clustered standard errors were estimated by facility. Each panel of the graph represents a different response variable on the *y*-axis with each *x*-axis giving the year relative to the year of sale (years leading or lagging the sale). Due to the variable year of the CHOW, only 10 NHs have data 6 years prior to the CHOW, and five NHs have data 5 years post-CHOW. The remaining distribution of NHs in time relative to the CHOW year is given under Figure 1. Dots on the figure give the regression coefficient estimates for the relative leading and lagging years and lines give the 95% confidence intervals. The dashed horizontal and vertical lines mark a coefficient estimate of 0 and time 0 (year of sale), respectively. The solid horizontal line gives the difference between 2016 mean response of constant ownership facilities and the intercept of the regression model averaged across all CHOW facilities.

Time differential two-way fixed-effects regression models were fit with the overall quality score and three subcomponents of the quality score as the response variables. Independent variables for each model were a binary indicator for post-CHOW years (difference-in-difference estimate), indicators for cost year, facility fixed effects, for-profit ownership, an indicator for the post-sale years for facilities that became for-profit as a result of the CHOW, and an interaction between for-profit ownership and post-VBR years (2016–2019). Changes in spending, utilization, and revenue patterns were assessed using the same regression models, but with the appropriate dependent variable. In all, 131 variables from the cost report were examined for changes associated with the CHOW. To adjust for multiple testing, *P*-values were inflated using the Benjamini–Hochberg adjustment (Benjamini & Hochberg, 1995).

## Results

Of the 80 NHs sold during the period, the majority of NHs either remained for-profit (68%) or became for-profit (21%) with a smaller number remaining not-for-profit (6%) or swapping between not-for-profit and government owned (5%). At least 51 of the NHs were sold to out of state buyers, 35 were sold to large chains, and 31 now leased the property from real estate investment trusts (data not available for all transactions).

Table 1 gives the comparison between all NHs that changed owners from 2014 to 2019 and those that had constant ownership over the same period. Comparisons are made in 2013 across a set of variables correlated with likelihood to change ownership. The CHOW NHs were more likely to be for-profit (68% vs. 16%). On a per resident day basis, CHOW NHs had less-activity staff, other direct care staff, and total nursing hours and had lower total dietary and plant costs. The CHOW NHs also had lower direct care staff retention rates particularly RN and social workers. Additionally, CHOW NHs had lower average quality of life scores than those NHs with constant ownership over the period.

**Table 1.** 2013 Means of Change of Ownership (CHOW) and Constant Ownership Group

Variable	CHOW between 2014–2019	Constant ownership 2014–2019
Number of nursing homes	80	262
*Nursing home characteristics*		
Ownership: for-profit	68%^a^	16%^a^
Urban twin cities	36%	27%
Occupancy and revenue		
Occupancy rate	88%	89%
Annual change in occupancy	−0.2%	−0.8%
Percent Medicaid revenue	51%	49%
Staffing characteristics		
Activities staff hours PRD	0.23^a^	0.29^a^
Other DC staff hours PRD	0.016^a^	0.045^a^
Total nursing hours PRD	4.41^a^	4.72^a^
Direct care staff retention	66%^a^	71%^a^
RN retention	61%^a^	72%^a^
SW retention	65%^a^	77%^a^
Compensation patterns		
LPN salary PRD	15.54	15.61
CNA salary PRD	29.57	31.32
Group medical PRD	7.12	6.84
% of salary on direct care salary	69%^a^	66%^a^
Costs		
Dietary total cost PRD	12.29^a^	13.98^a^
Plant total costs PRD	0.023^a^	0.030^a^
Quality indicator (max 50)	33.3	34.0
Quality of life (max 40)	32.8^a^	33.2^a^
MN Department of Health Inspection (Max 10)	8.3	8.5

*Note*s: DC = direct care, PRD = per resident day, RN = registered nurse, SW = social worker, LPN = licensed practical nurse, CNA = certified nursing assistant. Table includes the quality score components and variables that were correlated with change of ownership.

^a^Benjamini–Hochberg adjustment for multiple testing *P*-value < .05 (column 2 vs. 3).

Figure 1, the event study graphs, displays the average within facility trends of those NHs that changed ownership during the period after controlling for time and facility fixed effects. The majority of confidence intervals contain zero, indicating that most trends are not statistically significant. The top left panel shows that for these facilities, the overall VBR quality score was generally declining until the year of the sale and saw additional decline 3 and 4 years after the sale. The top right panel shows that the clinical quality indicator score, although somewhat higher prior to the sale, had a relatively high degree of variability such that a clear trend is not present. The bottom left panel shows that the quality of life scores were higher prior to the sale year and saw a significant decline in years 4 and 5 post-sale. In the bottom right panel, the Minnesota Department of Health inspection scores declined until 2 years prior to the sale, remaining stable through 1 year post-sale, before declining in years 2–4 post-sale.

The two-way fixed-effects model results are given in Table 2. Each column gives the model coefficients for a different response: total VBR score, clinical quality indicator score, quality of life score, and the Minnesota Department of Health inspection score. The model controls for individual facility differences through a facility fixed-effect term and time differences through cost year indicator variables, with 2015, the year just prior to the start of VBR as the baseline year. Additionally, the model controls for the financial organization of the facility with a “for profit” indicator and includes an interaction term between “for profit” status and the VBR implementation years to test for differential reaction to the policy. The primary variable of interest is the “Post-CHOW” term that measures the change in scores following a change in ownership. Of secondary interest is the indicator term that breaks out the post CHOW performance of the subset of NHs that became for-profit as a result of the CHOW (i.e., were nonprofit or government owned prior to the CHOW event).

**Table 2.** Difference-in-Difference Estimates for Various Measures of Quality

Independent variable	VBR score	Clinical quality indicators (max 50)	Quality of life surveys (max 40)	Minnesota Department of Health Inspections (max 10)
Post-CHOW^a^	−1.51*	−0.54	−0.22^+^	−0.75*
For-profit	−2.64	−2.26	−0.47	0.08
Became for-profit, post-CHOW	1.18	−0.24	0.14	1.26
For-profit in VBR period	0.16	0.68	−0.05	−0.47
2013	−1.30*	−1.38*	0.01	0.04
2014	−0.59^+^	−1.22*	0.02	0.57*
2016	0.71*	0.94*	−0.37*	0.10
2017	1.11*	1.83*	−0.67*	−0.05
2018	−1.90*	−1.03*	−0.43*	−0.52*
2019	−2.73*	−0.91*	−0.63*	−1.21*

*Notes*: VBR = value-based reimbursement. *N* = 342. Each column gives the total score or a component of the 100-point quality score used in the Value-Based Reimbursement Policy that is the response of the time differential two-way fixed-effects difference-in-difference model. The reference level for the year dummy variables is 2015. NH that became for-profit following the CHOW were either nonprofit or government owned prior to the CHOW.

*P-value < .05.

^+^ *P*-value < .10.

^a^Difference-in-difference estimate.

Total VBR scores saw a statistically significant drop of 1.51 points for NHs that experienced a change of ownership. The largest drop of component scores that make up the VBR score was in the Minnesota Department of Health inspection scores, which dropped by a statistically significant 0.75 points. NHs that became for-profit as a result of the CHOW outperformed their peers in the post period, but the difference was not statistically significant. For-profit facilities averaged a lower clinical quality indicators score (2.26 points lower, not significant), although some of that gap was erased during the VBR period (0.68 increase, not significant). Over time, the mean VBR score increased steadily until 2017, after which the mean fell below pre-VBR levels. This drop in scores was driven by a drop in mean clinical quality indicator scores and Minnesota Department of Health inspection scores. At least part of the drop in inspection scores was due to a change in scoring implemented by the health department in 2017.

Table 3 presents the NH characteristics, revenue sources, and expenditures that changed significantly among CHOW NHs post-sale relative to the control group as measured by the time differential two-way fixed-effects model. CHOW NHs saw a drop in occupancy rates of 4%, including an average drop in annual admissions by 40 residents. CHOW NHs saw a significant increase in revenue from Medicare Part B, an average increase of $35,000 per year. These NHs increased administrative management fee costs by $54,000 on average and had small increases in the number of social worker hours (295 per year) and social worker salary ($9,500 per year). CHOW NHs cut spending on direct care trainer salaries ($6,500 per year) and made cuts in employee dental ($4,800 per year) and medical ($15,900 per year) benefits. Additionally, these NHs reduced the number of part time employees by an average of 15 positions following the sale.

**Table 3.** Nursing Home Characteristics and Spending Patterns that Changed Significantly With New Ownership

Dependent variable	Coefficient	*P*-value^a^	% Change from pre- to post-sale^b^
Occupancy rate	−4%	.000	
Number of annual admissions	−40.4	.000	−13%
Medicare part B revenue per resident day	1.63	.000	215%
Number of part time employees	−15.1	.000	−44%
Direct care trainer salary per resident day	−0.52	.000	−37%
Social worker hours per resident day	0.02	.035	9%
Social worker salary per resident day	0.39	.047	12%
Administrative management fees per resident day	2.34	.016	26%
Group dental insurance per resident day	−0.18	.000	−66%
Group medical insurance per resident day	−2.31	.003	−8%

*Notes: N* = 342. Statistically significant differences due to change of ownership were tested using a time differential two-way fixed-effects difference-in-difference model with the row variable as the dependent variable. One hundred and thirty-one variables from the cost report were examined with nonsignificant changes, after the Benjamini–Hochberg adjustment, omitted from the table.

^a^ *P*-values presented are inflated using the Benjamini–Hochberg procedure to adjust for multiple testing.

^b^% Change is calculated on the raw dollar totals to avoid confounding per resident day figures with falling occupancy rates (i.e., constant spending with falling occupancy can give a positive coefficient). Change is between year prior to and year following the year of the ownership change.

## Discussion

This study examined the impact of NH ownership change on care quality within the context of VBR policy implementation in Minnesota. The policy’s improved NH cost coverage for Medicaid stays coincided with an uptick in the number of NHs that changed ownership. Patterns in the event study graphs indicating that quality of life and health department inspection scores were in decline prior to the sale suggesting that sellers may have viewed VBR an ideal time to offload the NH rather than trying to address declining quality. Both the overall VBR quality score and the health inspection score were found to have declined following the sale relative to the constant ownership group in the statistical model. There is some variability within the CHOW group with some CHOW NHs performing relatively well despite the general decline in performance of the group. Nonetheless, these results are concerning because these facilities were not able to maintain the basic standards of care required by the Health Department regulations. Two questions follow naturally from these findings: why did the drop in care quality (e.g., health inspection measures) occur and what, if anything, should be done about it?

### Changes in Expenditure and Decline in Care Quality

Examining differences in staffing and spending patterns between the pre- and post-sale periods provides some insight into potential changes in strategy that may be leading to reduced quality scores. Contextually important in understanding these differences is that occupancy rates in the CHOW NHs fell by four percentage points more than the control NHs. A drop in occupancy could be driven by problems that were occurring prior to the sale that may have motivated the sale; or they may have been a consumer response to uncertainly about new owners and the quality of their care; or both. Another impact of occupancy declines was loss of revenue from unfilled beds. However, the CHOW group increased revenue from Medicare part B by a factor of 2.15 ($1.63 per resident day) which may be indicative of a strategy to supplement lost revenue from falling census by expanding potentially more profitable Medicare revenue. Although the cost report does not breakdown the particular activities that generate Medicare part B revenue for the NHs, relevant common activities in this category include outpatient physical therapy, outpatient speech-language pathology services, and outpatient occupational therapy. Likely due to the structure of the new ownership groups, CHOW NHs increased the amount spent on administrative management fees or typically money paid to the corporate headquarters for centralized services which range from a full management contract to individual items such as billing and collection, payroll services, accounting services, and information technology services and support. Tracing these fees through the corporate structure is difficult as is determining if increased expenditures result in better or more efficient management. Lastly, it appears that the CHOW group reduced expenditures by cutting staff medical and dental benefits and cutting an average of 15 part time positions.

Our findings indicate that a change in ownership has potential to negatively impact care quality. Further research is needed to delineate the causal pathways leading to lower quality care. First, it is possible that the organizational disruption that occurs when an ownership change occurs impacts daily facility function in the short term, decreasing performance on quality indicators. A strength of our study is the longitudinal design. However, following the CHOW facilities further in time past the CHOW event may provide insights into the contribution of an organizational disruption on level of quality. The finding that CHOW facilities had lower staff retention is possibility supportive of the idea that the change event caused short-term gaps in care. Second, it is quite possible that CHOW facilities were experiencing an unmeasured potential decline that impacted the seller’s desire to release the facility, and perhaps impacted for-profit or private equity owners’ decision to enter the market. The event study charts in Figure 1 suggest unexpectedly higher quality scores prior to the sale that support this possibility. Additionally, there is evidence that occupancy was declining prior to sale in many CHOW facilities, and further analysis of market level characteristics may illuminate the motivation to buy and sell particular facilities in a way that provides explanatory evidence for the decline in quality. Qualitative investigations, such as interviews with staff who can describe the organizational culture, context, and the impact of change on their work and observed level of quality, is a necessary next step.

### Limitations

The difference-in-difference approach is meant to approximate experimental conditions through the use of the natural experimental framework. Nonetheless, it is still a quasiexperimental design and causal inference from our results should be made with care. The downwards trend in quality of the CHOW facilities prior to sale make it difficult to distinguish how much blame for poor quality is assignable to the prior owners or to the current owners. However, that their tends to be a drop in quality scores associated with a CHOW suggests that policy designed to give clear expectations to new NH owners may better serve the residents of these NHs.

### Recommendations

What might be done to combat decline in NH care quality due to change of ownership? Given the national trends of ownership by large for-profit chains and private equity firms, a natural mechanism would be to strengthen the link between the profit incentive and the quality incentive of VBR. The current quality incentive is too weak to expect much of a reaction from NHs (Arling et al., 2019). Strengthening the quality incentive to put more of the NH revenue at risk may serve both to motivate self-correction among poor performers and to deter potential new owners who are not willing to fully commit to pursuing a high standard of care quality. A second mechanism would be to tie issuance of operating licenses to quality performance criteria that must be met while operating under an initial provisional license. The use of provisional licensing would give new ownership time to make needed changes to meet the minimum quality required for licensure. Some care may be needed to make sure both the property owner and the operator are incentivized to meet quality metrics by working together as they are sometimes two separate entities. To prepare for potential legal challenges from license denials, clear performance guidelines must be established. Adding complication to enforcement of policies linked to ownership are the relatively loose ownership documentation laws in the United States. Therefore, the licensure policy should seek to avoid creating a loophole that incentivizes the creation of a “new” owner or operator that fronts the purchase for a poor performing owner or operator. Given the changing landscape of ownership structures in the NH industry, and the influx of new owners and the associated decline in quality scores in the state, regardless of the nature of the policy adjustment, it is important VBR remain current to insure a NH industry that is characterized by person-centered care.
